# The association between fluid balance trajectories and prognosis in ICU patients with cardiac arrest, a group-based trajectory model analysis

**DOI:** 10.3389/fnut.2025.1664640

**Published:** 2025-09-01

**Authors:** Qitian Zhang, Guangyu Lin, Chunmei Zhang

**Affiliations:** Department of Cardiology, Zhangzhou Affiliated Hospital of Fujian Medical University, Zhangzhou, Fujian, China

**Keywords:** cardiac arrest, fluid balance, group-based trajectory model, MIMIC-IV database, prognosis

## Abstract

**Background:**

The impact of dynamic fluid balance (FB) changes on the prognosis of ICU patients with cardiac arrest (CA) remains unclear. This study aims to explore the association between FB trajectories and the prognosis of such patients.

**Methods:**

Data were sourced from CA patients in the MIMIC-IV database. A Group-Based Trajectory Model (GBTM) was used to identify patient subgroups with similar FB trajectories. Kaplan–Meier survival curves and Cox regression models were applied to analyze the association between FB trajectories and survival outcomes in CA patients. Subgroup and sensitivity analyses were conducted to further validate the robustness of the results.

**Results:**

A total of 876 CA patients were included. Four distinct FB trajectory patterns were identified, Trajectory 1 (rapid transition to negative balance), Trajectory 2 (stable balance), Trajectory 3 (positive balance gradually decreasing), and Trajectory 4 (decreasing at a high level). Kaplan–Meier survival analysis showed that the survival rate in Trajectory 1 was significantly higher than in the other trajectory groups, with the fluid overload group exhibiting a notably higher mortality risk than the non-overload group. Cox proportional hazards analysis indicated that, after adjusting for various covariates, the survival rate in Trajectory 1 remained significantly higher than in other trajectory groups (Reference, Trajectory 1; Trajectory 2, HR = 1.75 [1.31–2.34], Trajectory 3, HR = 2.02 [1.53, 2.68], Trajectory 4, HR = 1.71 [1.24, 2.37]). Subgroup and sensitivity analyses did not alter these findings.

**Conclusion:**

The GBTM method helps to identify subgroups of ICU cardiac arrest patients with distinct risk profiles. Among the dynamic FB types, the group with rapid transition to negative balance at a moderate level (Trajectory 1) showed the best prognosis.

## 1 Introduction

Cardiac arrest (CA) refers to the sudden cessation of heart function, leading to the interruption of blood circulation (1). It is a significant public health issue. In the United States, over 600,000 people are affected by CA annually, with a global incidence rate of 30 to 97 cases per 100,000 people (2–4). The overall survival rate for CA patients remains low. The out-of-hospital survival rate for adult patients after CA resuscitation is 9%, while the in-hospital survival rate is 23% (4). Even among those who survive to discharge, the risk of death significantly increases during the subsequent course, with the global 1-year survival rate for CA patients being only 7.7% (5). Intensive care management for CA survivors is critical, as it directly impacts both survival rates and neurological outcomes (6).

Following cardiac arrest, a series of complex pathophysiological processes commonly occur, including ischemia–reperfusion injury, activation of the inflammatory cascade, and organ dysfunction, often resulting in a high incidence of post-resuscitation shock (6). Current post-cardiac arrest care guidelines offer general recommendations for hemodynamic management, but evidence on optimal fluid therapy remains limited (7). Early studies have indicated that excessive positive fluid balance (FB) during ICU stays is associated with poor prognosis in critically ill patients (8). Despite the development of various strategies to assess fluid responsiveness (9), the optimal fluid management regimen remains a topic of controversy. A multicenter study found that positive FB following out-of-hospital CA was significantly associated with poor outcomes, and a restrictive fluid management strategy may be beneficial for CA patients (10). Moreover, several studies have shown that in patients receiving extracorporeal cardiopulmonary resuscitation (ECPR) after CA, positive FB is associated with adverse outcomes (8, 11). These studies on FB were based on fluid status at specific time points and failed to capture the dynamic changes in overall FB. Group-Based Trajectory Modeling (GBTM) can more precisely describe and understand individual responses heterogeneity, and it can be used to explore dynamic changes over time (12). GBTM has been widely applied in medical, psychological, and other fields (13).

This study aims to explore the association between FB changes and prognosis in ICU patients with CA. We will use the GBTM approach to analyze FB trajectories in CA patients and examine the relationship between different trajectory patterns and 30-day survival. The findings of this study could help optimize fluid management strategies for CA patients, enabling the precise identification of high-risk groups, which may ultimately help conserve medical resources and reduce uncertainty during critical periods.

## 2 Materials and methods

### 2.1 Data source

The data for this study were sourced from the MIMIC-IV (version 3.1) database (14). This is a publicly available de-identified dataset that includes comprehensive health records of patients admitted to the ICU at Beth Israel Deaconess Medical Center between 2008 and 2022. All data in the dataset have been de-identified, and therefore, informed consent from patients is not required. Additionally, approval from an institutional review board or ethics committee is not needed. The study adhered strictly to the guidelines outlined in the Strengthening the Reporting of Observational Studies in Epidemiology (STROBE) statement for observational studies (15).

### 2.2 Study population

We selected patients with CA from the MIMIC-IV database. CA patients were identified using ICD-9 codes (4275) and ICD-10 codes (I46, I462, I468, I469). The inclusion criteria were, (1) age > 18 and <100 years; (2) first ICU admission; (3) ICU stay ≥ 3 days. The exclusion criteria were, (1) fewer than 3 FB measurements within 7 days; (2) missing data exceeding 10%; (3) missing body weight at admission.

### 2.3 Variables and outcomes

We extracted FB data and prognosis indicators for the first 1 to 7 days after ICU admission for CA patients. Additional variables collected included, (1) demographic data, age, sex, body weight; (2) vital signs, temperature, heart rate, respiratory rate, blood pressure; (3) laboratory results, complete blood count, blood biochemistry, coagulation profile, blood gas analysis, etc.; (4) comorbidities, acute myocardial infarction, atrial fibrillation, hypertension, diabetes, hyperlipidemia, chronic obstructive pulmonary disease, pneumonia, chronic kidney disease; (5) medications, ACE inhibitors/ARBs, beta-blockers, diuretics, and vasopressors; (6) other variables, use of CRRT, mechanical ventilation, sepsis, acute kidney injury, and severity scores (SOFA, Charlson score). Data were extracted using Navicat Premium 17.0 software and structured query language (SQL) from the MIMIC-IV database, recording fluid input and output data from Day 1 to Day 7. We summarize and calculate the daily fluid balance based on the data from the inputevents and outputevents tables, using timestamps. Laboratory indicators were extracted as average values within the first 24 h of ICU admission.

FB was calculated using the formula, FB = (Total fluid input - Total fluid output)/Initial body weight (kg). Fluid overload (FO) was defined as cumulative FB exceeding 10% of initial body weight (16). The primary endpoint was the 30-day in-hospital mortality, defined as the survival status of CA patients within 30 days of ICU admission.

### 2.4 Group-based trajectory modeling

Group-Based Trajectory Modeling (GBTM) is a semi-parametric model designed for longitudinal data analysis, capable of identifying FB trajectory patterns within the same cohort (17). In this study, the GBTM method was used to fit FB trajectories using a cubic polynomial, with the optimal number of groups determined by model parameters. After selecting the number of groups, the significance of linear, quadratic, and cubic polynomials was assessed to optimize trajectory shapes. The criteria for selecting the best trajectory included, (1) Bayesian Information Criterion (BIC) and Akaike Information Criterion (AIC), values closer to 0 indicate better fit; (2) Average posterior probability (Avepp), >0.7 suggests reliable subgroup classification; (3) Proportion of patients in each trajectory group >5%; (4) OCC (correct classification rate), the minimum OCC value should be greater than 5.0, with higher values indicating better classification; (5) A comprehensive evaluation of model simplicity and clinical interpretability.

### 2.5 Statistical analysis

In baseline description, normality testing of continuous variables was performed using the Shapiro–Wilk test. Normally distributed data were presented as mean ± standard deviation (SD), and between-group comparisons were performed using one-way ANOVA. Non-normally distributed data were expressed as median (interquartile range), and comparisons between groups were conducted using the Kruskal-Wallis test. Categorical variables were presented as frequencies and percentages, and comparisons were made using the Chi-squared (χ2) test or Fisher’s exact test. Samples with more than 10% missing data were excluded. Since the missing data is primarily missing at random and based on the correlation between variables, we used the KNN method to impute the missing values. Kaplan–Meier curves were used to compare survival differences between different FB trajectory groups. Univariate and multivariate forward stepwise Cox regression analyses were conducted to assess the association between trajectory groups and study outcomes, with a threshold for inclusion set at 0.1 and exclusion at 0.05. A correlation heatmap was generated to analyze the relationships between variables, and the variance inflation factor (VIF) was calculated to test for multicollinearity between covariates. Variables with a correlation coefficient >0.5 or VIF > 5 were further filtered. Cox proportional hazards models were used to calculate hazard ratios (HRs) and 95% confidence intervals (CIs) to assess the relationship between FB trajectories and 30-day mortality in CA patients. Subgroup analyses were performed to explore potential influencing factors between FB groups and prognosis in CA patients, including age (≤65 vs. > 65 years), sex (male vs. female), and SOFA score (<6 vs. ≥ 6). Additionally, a sensitivity analysis excluding patients who received CRRT was conducted to verify the robustness of the results. Statistical analysis was performed using R software (version 4.4.3, www.r-project.org), with a two-sided *p* value < 0.05 considered statistically significant.

## 3 Results

### 3.1 Baseline characteristics

**Figure 1:**
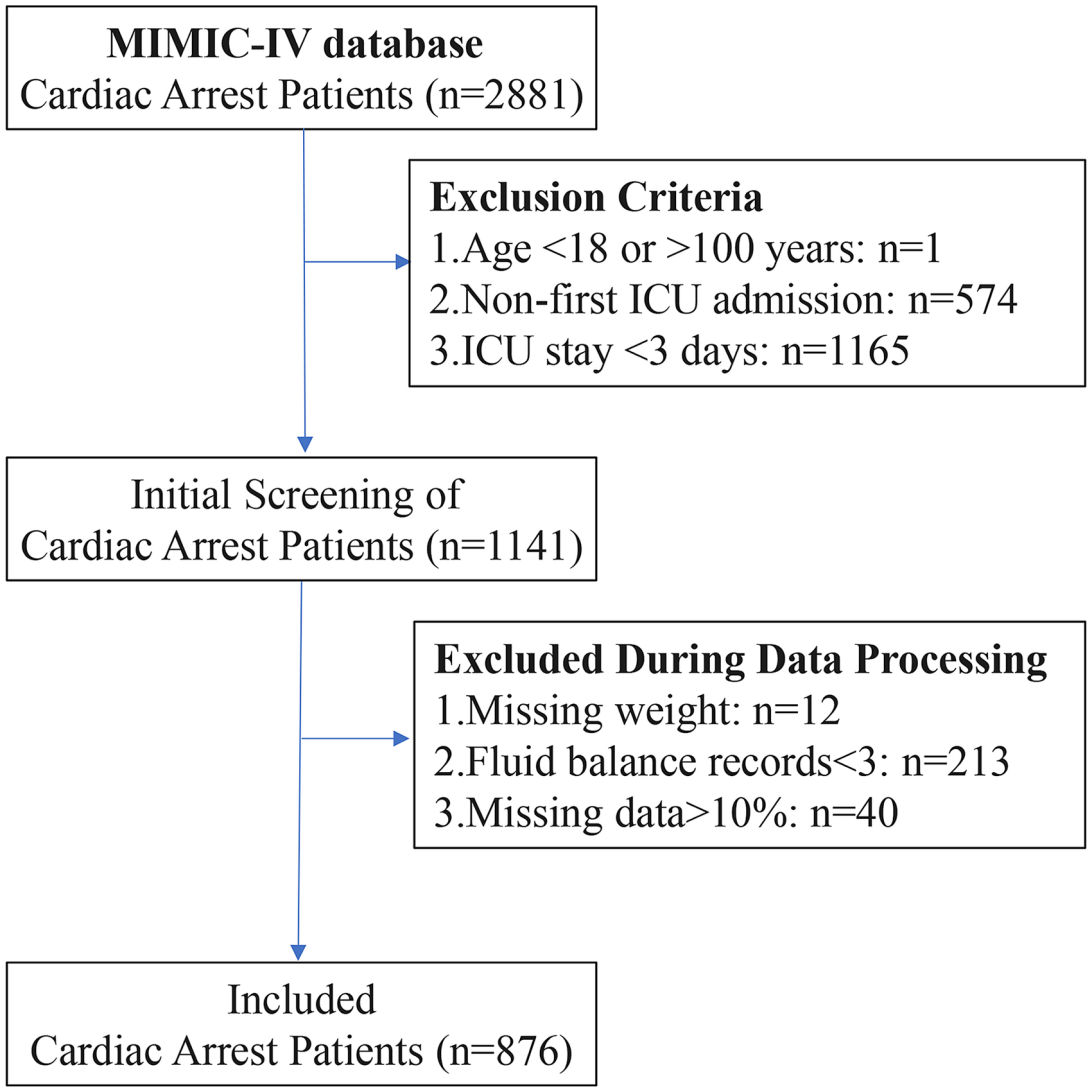
Flowchart of CA patient selection process.

A total of 2,881 patients with CA were selected from the MIMIC-IV database. After applying the exclusion criteria, 876 CA patients were included in the final study cohort (Figure 1). The variable screening results are shown below, including missing data (Supplementary Figure 1), high correlation (Supplementary Figure 2), and strong collinearity (Supplementary Figure 3).

Table 1 presents the baseline characteristics of the patients. The median age of all CA patients was 67.0 years, with 555 (63.4%) being male. A total of 132 (15.1%) patients received continuous renal replacement therapy (CRRT), 199 (22.7%) patients had acute myocardial infarction, and 335 (38.2%) patients had atrial fibrillation. The average SOFA score at ICU admission was 8.0, and the average Charlson comorbidity score was 5.0.

**Table 1:** Baseline characteristics of CA patients with different fluid balance trajectory groups.

Variables	Total (*n* = 876)	1 (*n* = 133)	2 (*n* = 298)	3 (*n* = 313)	4 (*n* = 132)	*p*
Demographics						
Age (years)	67.0 [56.0; 78.0]	69.0 [58.0; 81.0]	67.0 [56.0; 76.0]	67.0 [56.0; 79.0]	67.5 [56.0; 81.0]	0.341
Gender						0.028
Female	321 (36.6%)	50 (37.6%)	93 (31.2%)	117 (37.4%)	61 (46.2%)	
Male	555 (63.4%)	83 (62.4%)	205 (68.8%)	196 (62.6%)	71 (53.8%)	
Weight (kg)	81.1 [69.1; 95.5]	81.0 [70.8; 95.4]	86.0 [75.7; 100.2]	79.5 [67.0; 95.0]	72.6 [60.0; 85.2]	<0.001
Vital signs						
T (°C)	36.8 [36.4; 37.2]	36.8 [36.4; 37.2]	36.8 [36.5; 37.1]	36.8 [36.4; 37.2]	36.7 [36.3; 37.0]	0.029
HR (bpm)	89.0 [75.0; 105.0]	84.0 [73.0; 103.0]	87.0 [72.0; 101.0]	90.0 [75.0; 106.0]	96.0 [82.8; 115.0]	<0.001
RR (bpm)	19.0 [16.0; 24.0]	19.0 [15.0; 23.0]	20.0 [16.0; 23.0]	20.0 [16.0; 25.0]	19.0 [16.0; 24.2]	0.422
SBP (mmHg)	122.0 [103.0; 140.0]	115.0 [99.0; 136.0]	125.0 [107.0; 142.8]	124.0 [106.0; 143.0]	111.0 [97.8; 133.0]	<0.001
SpO_2_ (%)	99.0 [96.0; 100.0]	100.0 [97.0; 100.0]	99.0 [96.0; 100.0]	99.0 [96.0; 100.0]	99.0 [95.0; 100.0]	0.176
Laboratory indicators						
WBC (m/uL)	13.0 [8.6; 18.2]	13.1 [9.1; 18.2]	12.7 [8.7; 17.5]	12.6 [8.5; 18.1]	13.7 [8.2; 19.5]	0.633
PLT (K/uL)	197.0 [141.0; 259.0]	194.0 [138.0; 244.0]	200.5 [157.0; 266.2]	192.0 [134.0; 257.0]	189.0 [137.8; 263.0]	0.101
Hb (g/dL)	11.0 [9.1; 13.0]	11.0 [8.6; 13.0]	11.3 [9.2; 13.3]	10.8 [9.0; 12.8]	10.9 [9.1; 12.7]	0.192
Na (mEq/L)	139.0 [136.0; 142.0]	139.0 [137.0; 142.0]	139.0 [135.0; 141.0]	139.0 [136.0; 142.0]	140.0 [136.0; 143.0]	0.078
K (mEq/L)	4.2 [3.7; 4.7]	4.1 [3.7; 4.6]	4.2 [3.8; 4.7]	4.2 [3.7; 4.7]	4.2 [3.7; 4.8]	0.736
Ca (mg/dL)	8.3 [7.7; 8.7]	8.3 [7.8; 8.7]	8.4 [7.9; 8.8]	8.3 [7.6; 8.7]	7.8 [7.4; 8.4]	<0.001
GLU (mg/dL)	160.2 [121.8; 220.2]	156.0 [123.0; 212.0]	160.8 [120.0; 220.0]	159.0 [124.0; 212.0]	163.5 [120.8; 242.8]	0.747
AG (mEq/L)	16.0 [13.0; 19.0]	15.0 [13.0; 18.0]	16.0 [13.0; 19.0]	16.0 [13.0; 19.0]	17.0 [13.8; 22.0]	0.021
ALT (IU/L)	54.0 [26.0; 100.0]	45.0 [23.0; 71.0]	54.0 [27.0; 122.8]	54.0 [28.0; 103.0]	43.0 [21.0; 71.0]	0.011
Cr (mg/dL)	1.2 [0.9; 1.7]	1.1 [0.8; 1.4]	1.2 [0.9; 1.8]	1.2 [0.8; 1.7]	1.2 [1.0; 1.6]	0.08
PT (sec)	14.5 [12.6; 17.2]	14.5 [12.9; 18.0]	13.9 [12.3; 16.2]	14.5 [12.5; 17.1]	15.1 [13.6; 18.0]	0.001
APTT (sec)	32.7 [28.0; 37.8]	32.7 [29.0; 36.7]	31.5 [27.8; 37.8]	32.5 [27.7; 37.6]	32.7 [28.5; 39.2]	0.201
pH (units)	7.3 [7.2; 7.4]	7.3 [7.2; 7.4]	7.3 [7.2; 7.4]	7.3 [7.2; 7.4]	7.3 [7.2; 7.3]	<0.001
PaO_2_ (mmHg)	100.0 [55.0; 208.0]	119.0 [62.0; 291.0]	90.0 [50.0; 166.8]	99.0 [59.0; 213.0]	116.0 [61.0; 239.0]	0.002
Lac (mmol/L)	2.5 [1.6; 4.2]	2.7 [1.8; 4.3]	2.1 [1.4; 3.4]	2.6 [1.7; 4.4]	2.6 [1.9; 5.1]	<0.001
Comorbidities						
AMI	199 (22.7%)	26 (19.5%)	95 (31.9%)	53 (16.9%)	25 (18.9%)	<0.001
Afib	335 (38.2%)	53 (39.8%)	114 (38.3%)	110 (35.1%)	58 (43.9%)	0.357
HTN	603 (68.8%)	97 (72.9%)	206 (69.1%)	215 (68.7%)	85 (64.4%)	0.518
DM	317 (36.2%)	50 (37.6%)	114 (38.3%)	113 (36.1%)	40 (30.3%)	0.449
HLP	326 (37.2%)	55 (41.4%)	121 (40.6%)	105 (33.5%)	45 (34.1%)	0.188
COPD	51 (5.8%)	6 (4.5%)	25 (8.4%)	17 (5.4%)	3 (2.3%)	0.068
PNA	262 (29.9%)	34 (25.6%)	105 (35.2%)	82 (26.2%)	41 (31.1%)	0.061
CKD	228 (26.0%)	31 (23.3%)	79 (26.5%)	85 (27.2%)	33 (25.0%)	0.843
Drugs						
ACEI ARB	213 (24.3%)	46 (34.6%)	85 (28.5%)	64 (20.4%)	18 (13.6%)	<0.001
Beta Blocker	598 (68.3%)	98 (73.7%)	217 (72.8%)	199 (63.6%)	84 (63.6%)	0.028
Furosemide	639 (72.9%)	116 (87.2%)	227 (76.2%)	201 (64.2%)	95 (72.0%)	<0.001
Spironolactone	50 (5.7%)	12 (9.0%)	21 (7.0%)	15 (4.8%)	2 (1.5%)	0.037
Dobutamine	60 (6.8%)	11 (8.3%)	16 (5.4%)	21 (6.7%)	12 (9.1%)	0.476
Dopamine	132 (15.1%)	18 (13.5%)	34 (11.4%)	62 (19.8%)	18 (13.6%)	0.028
Epinephrine	181 (20.7%)	44 (33.1%)	41 (13.8%)	58 (18.5%)	38 (28.8%)	<0.001
Norepinephrine	617 (70.4%)	95 (71.4%)	180 (60.4%)	233 (74.4%)	109 (82.6%)	<0.001
Phenylephrine	357 (40.8%)	60 (45.1%)	73 (24.5%)	141 (45.0%)	83 (62.9%)	<0.001
Other indicators						
Ventilation	824 (94.1%)	128 (96.2%)	277 (93.0%)	296 (94.6%)	123 (93.2%)	0.549
CRRT	132 (15.1%)	4 (3.0%)	31 (10.4%)	64 (20.4%)	33 (25.0%)	<0.001
Sepsis	792 (90.4%)	119 (89.5%)	263 (88.3%)	286 (91.4%)	124 (93.9%)	0.266
AKI	859 (98.1%)	128 (96.2%)	293 (98.3%)	308 (98.4%)	130 (98.5%)	0.478
SOFA	8.0 [5.0;11.0]	9.0 [6.0;11.0]	7.0 [4.0;9.0]	8.0 [6.0;11.0]	10.5 [8.8;13.0]	<0.001
Charlson	5.0 [3.0;8.0]	5.0 [3.0;7.0]	5.0 [3.0;8.0]	5.0 [3.0;8.0]	5.0 [3.0;8.0]	0.97

T, Temperature; HR, Heart Rate; RR, Respiratory Rate; SBP, Systolic Blood Pressure; SpO₂, Peripheral Capillary Oxygen Saturation; WBC, White Blood Cell; PLT, Platelet; Hb, Hemoglobin; Na, Sodium; K, Potassium; Ca, Calcium; GLU, Glucose; AG, Anion Gap; ALT, Alanine Aminotransferase, Cr, Creatinine; PT, Prothrombin Time; APTT, Activated Partial Thromboplastin Time; pH, Hydrogen Ion Concentration; PaO₂, Partial Pressure of Oxygen; Lac, Lactate; AMI, Acute Myocardial Infarction; Afib, Atrial Fibrillation; HTN, Hypertension; DM, Diabetes Mellitus; HLP, Hyperlipidemia; COPD, Chronic Obstructive Pulmonary Disease; PNA, Pneumonia; CKD, Chronic Kidney Disease; ACEI ARB, Angiotensin-Converting Enzyme Inhibitor / Angiotensin II Receptor Blocker; Beta Blocker, Beta-Adrenergic Blocking Agent; CRRT, Continuous Renal Replacement Therapy; AKI, Acute Kidney Injury; SOFA, Sequential Organ Failure Assessment; Charlson, Charlson Comorbidity Index.

### 3.2 FB trajectory description

Before grouping the FB trajectories, we initially examined FB from Day 1 to Day 7. However, due to a high proportion of missing data, the missing rates from Day 4 to Day 7 were 5.4, 21.9, 33.9, and 43.9%, respectively. Due to substantial missing data on Days 5–7, analysis was limited to FB from Days 1 to 4. Table 2 presents the model fit statistics and average posterior probabilities (AvePP) used to determine the optimal number of FB trajectory groups. The AIC and BIC for 4-group and 5-group models were similar, with the smallest values in all models. Furthermore, the proportion of the population in each group exceeded 5%, and both AvePP and the minimum OCC (correct classification rate) met the required thresholds. After considering the overall interpretability and simplicity of the model, we selected the four-group trajectory model.

**Table 2:** Performance of the group-based trajectory model for fluid balance trajectories.

Trajectories	BIC	AIC	AvePP	Minimum OCC	Class proportion
1 group	33362.98	33338.33	1.00	NaN	100.0%
2 groups	32641.99	32580.38	0.93/0.90	6.17	70.4%/29.6%
3 groups	32537.04	32444.62	0.86/0.79/0.89	4.85	55.0%/32.4%/12.6%
4 groups	32504.26	32368.70	0.75/0.78/0.78/0.88	6.60	14.0%/34.5%/35.7%/15.7%
5 groups	32445.72	32273.19	0.78/0.79/0.78/0.70/0.89	8.31	11.2%/35.7%/14.4%/22.6%/16.1%

BIC, Bayesian information criterion; AIC, Akaike information criterion; AvePP, average posterior probability; OCC, odds of correct classification.

**Figure 2:**
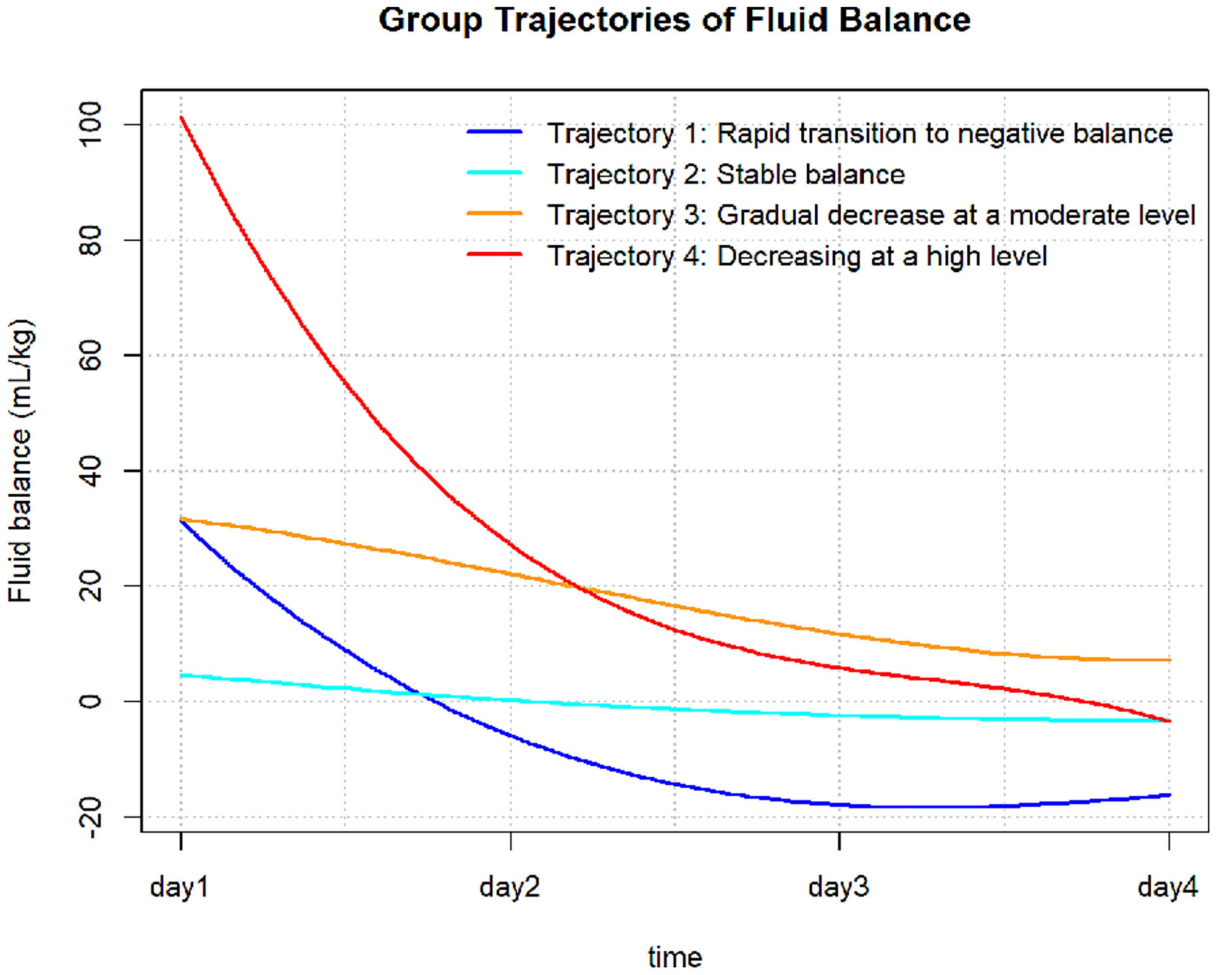
Fluid balance trajectories in patients with CA.

The FB trajectory groups are shown in Figure 2. Trajectory 1 (rapid transition to negative balance) included 133 (15.2%) patients, with an FB of approximately 30 mL/kg on Day 1, rapidly decreasing to a negative balance and maintaining around −20 mL/kg. Trajectory 2 (stable balance) included 298 (34.0%) patients, with an FB of approximately 0 mL/kg on Day 1, showing no significant fluctuations, remaining close to 0 mL/kg. Trajectory 3 (positive balance gradually decreasing) included 313 (35.7%) patients, with an FB of approximately 30 mL/kg on Day 1, slowly decreasing but remaining above 0 mL/kg each day. Trajectory 4 (high-level decrease) included 132 (15.1%) patients, with the highest FB on Day 1 at approximately 100 mL/kg, rapidly decreasing to near 0 mL/kg by Days 3 and 4. The baseline characteristics of each trajectory group are shown in Table 1. There were no significant differences between the trajectory groups regarding age, Charlson score, mechanical ventilation use, sepsis, or acute kidney injury (AKI). The proportion of patients with acute myocardial infarction was higher in Trajectory 2 compared to the other groups. However, no significant differences were observed for other comorbidities, such as atrial fibrillation, hypertension, diabetes, hyperlipidemia, COPD, and chronic kidney disease (CKD).

### 3.3 Relationship between FB trajectories, FO status, and survival

**Figure 3:**
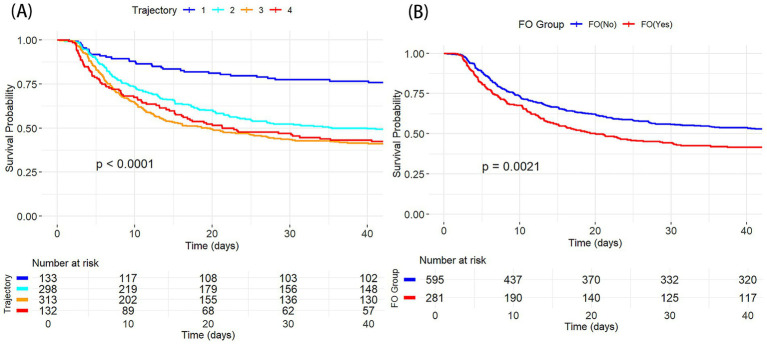
K-M survival curves showing the relationship between different CA groups and 30 day mortality. **(A)** By FB trajectory groups; **(B)** By FO status.

Kaplan–Meier survival analysis revealed significant survival differences between the FB trajectory groups and FO status (Figure 3). There was a significant difference in survival rates between the groups (*p* < 0.001), with Trajectory 1 (rapid transition to negative balance) having the best survival. Additionally, the mortality risk was significantly higher in the FO group compared to the non-overload group (*p* = 0.0021).

Univariate and multivariate Cox regression results are presented in Supplementary Table 1. Univariate analysis showed that trajectory group was significantly associated with 30-day mortality. Furthermore, multivariate Cox regression revealed that (Table 3), compared to Trajectory 1 (rapid transition to negative balance), other trajectory groups were significantly associated with increased 30-day mortality risk (Reference, Trajectory 1; Trajectory 2, HR = 1.73, 95% CI 1.29–2.32, *p* < 0.001; Trajectory 3, HR = 1.93, 95% CI 1.45–2.57, *p* < 0.001; Trajectory 4, HR = 1.61, 95% CI 1.16–2.24, *p* = 0.004). Other significant factors influencing 30-day mortality included the presence of atrial fibrillation, pneumonia, CKD, and use of ACE inhibitors/ARBs, beta-blockers, furosemide, mechanical ventilation, as well as age, weight, platelet count (PLT), prothrombin time (PT), PaO_2_, and Charlson score (all *p* < 0.05). After adjusting for various covariates, multivariate Cox regression was used to evaluate the relationship between FB trajectories and 30-day survival (Table 3). Model 1 did not adjust for covariates, while Model 2 adjusted for age and weight, Model 3 added PLT, PT, PaO_2_, and Charlson score, and Model 4 further adjusted for atrial fibrillation, pneumonia, CKD, ACE inhibitors/ARBs, beta-blockers, furosemide, and mechanical ventilation. In all Cox regression models, Trajectory 1 had the best survival. After adjusting for multiple confounding factors, Model 4 showed that the mortality risk in the other trajectory groups was higher than in Trajectory 1 (Trajectory 2, HR = 1.75 [1.31–2.34], Trajectory 3, HR = 2.02 [1.53, 2.68], Trajectory 4, HR = 1.71 [1.24, 2.37]).

**Table 3:** Cox regression multimodel analysis of fluid balance trajectories in CA patients.

Variables	Model 1		Model 2		Model 3		Model 4	
	HR (95%CI)	*p*	HR (95%CI)	*p*	HR (95%CI)	*p*	HR (95%CI)	*p*
Trajectory								
1	1.00 (Reference)		1.00 (Reference)		1.00 (Reference)		1.00 (Reference)	
2	1.78 (1.35 ~ 2.37)	**<0.001**	1.93 (1.46 ~ 2.57)	**<0.001**	1.84 (1.38 ~ 2.45)	**<0.001**	1.75 (1.31 ~ 2.34)	**<0.001**
3	2.33 (1.77 ~ 3.07)	**<0.001**	2.46 (1.86 ~ 3.25)	**<0.001**	2.38 (1.80 ~ 3.14)	**<0.001**	2.02 (1.53 ~ 2.68)	**<0.001**
4	2.01 (1.46 ~ 2.77)	**<0.001**	2.01 (1.46 ~ 2.77)	**<0.001**	1.93 (1.40 ~ 2.67)	**<0.001**	1.71 (1.24 ~ 2.37)	**0.001**

HR, Hazard Ratio; CI, Confidence Interval.

Model 1, Crude.

Model 2, Adjust, Age, Weight.

Model 3, Adjust, Age, Weight, PLT, PT, PaO_2_, Charlson.

Model 4, Adjust, Age, Weight, PLT, PT, PaO_2_, Charlson, AFib, PNA, CKD, ACEI.ARB, beta.blocker, Furosemide, Ventilation.

Bold values in Table 3 highlight p-values that reached statistical significance.

### 3.4 Subgroup analysis

**Figure 4:**
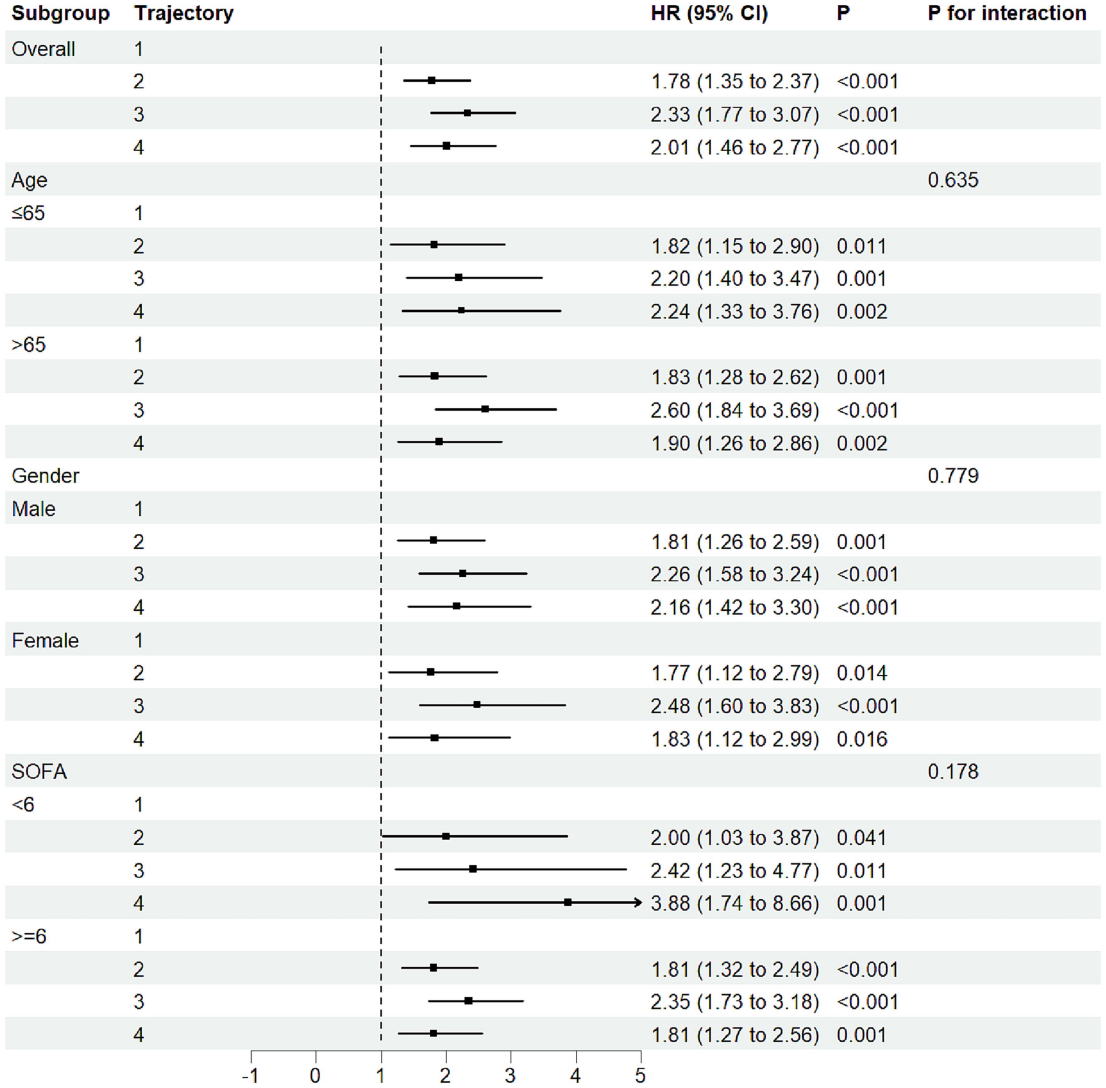
Forest subgroup analysis FB trajectories and 30-day ICU mortality in CA patients.

We performed stratified subgroup analyses based on age, sex, and SOFA score, with results shown in Figure 4. No significant interactions were found, indicating robustness across demographic subgroups (P for interaction > 0.05). These results suggest that the association between FB trajectory groups and 30-day mortality risk was consistent across different baseline characteristics in the study population.

### 3.5 Sensitivity analysis

In the sensitivity analysis, we excluded 132 (15.1%) CA patients who received CRRT to eliminate potential effects of CRRT on FB. Despite this exclusion, we observed similar FB trajectory patterns (Supplementary Figure 4). Of these, 137 (18.4%) patients were in the rapid transition to negative balance group, 231 (31.0%) in the stable balance group, 293 (39.4%) in the moderate decline group, and 83 (11.1%) in the high-level decrease group. The 30-day all-cause mortality rate was lowest in the rapid transition to negative balance group (*p* = 0.027) (Supplementary Figure 5), consistent with the results prior to the sensitivity analysis.

## 4 Discussion

This study utilized the GBTM method to explore the FB trajectory in CA patients, identifying a correlation between FB trajectory levels and mortality risk. We identified four distinct FB trajectories, trajectory 1 (rapid transition to negative balance), trajectory 2 (stable balance), trajectory 3 (positive balance gradually decreasing), and trajectory 4 (gradual decrease at high level). After adjusting for all confounding factors, it was observed that the 30-day mortality risk for trajectory 1 was significantly lower than that of the other trajectory groups. Subgroup and sensitivity analyses showed similar results. Additionally, we observed that FO was associated with an increased risk of mortality.

CA patients typically experience circulatory failure. This leads to a systemic inflammatory response, which includes pathological vasodilation, increased capillary permeability, and hypoalbuminemia. To maintain vascular content and improve cardiac output, large volumes of fluids and other medications are often required (18, 19). Post-resuscitation shock after out-of-hospital cardiac arrest is quite common, with an incidence of 50 to 70% (20, 21). Our study found that among the various FB trajectories, CA patients in the rapid negative balance group had a significantly lower 30-day ICU mortality rate. Several studies have reported similar findings. A multicenter study found that positive FB after out-of-hospital cardiac arrest was significantly associated with poor outcomes, with a dose-dependent relationship, suggesting that restrictive fluid therapy strategies might be beneficial for post-cardiac arrest patients (10). Multiple studies on post-cardiac arrest patients who underwent cardiopulmonary resuscitation have shown that early negative FB is associated with higher ICU survival rates (8, 11). For patients with cardiac arrest receiving venous–arterial extracorporeal membrane oxygenation (VA-ECMO) support, each additional liter of cumulative FB on day 7 increases the 28-day mortality risk by 11% (22). Although the results are similar, there are several points to note. First, our study focused on ICU patients with CA, primarily concerned with their survival, without detailed classification of CA patients. Second, other studies primarily focus on FB at a single time point after cardiac arrest, which cannot reflect the complex dynamic changes in FB. By classifying CA patients using the GBTM method, we are better able to capture these dynamic changes. Lastly, it is important to note that higher FB may reflect more severe illness, and the clinical judgment required for more fluid resuscitation (23). This confounding factor should be acknowledged as a limitation, as the severity of illness could influence the fluid management approach and affect our findings.

FO has been shown to be harmful to critically ill patients of various types (24, 25). Several pathophysiological mechanisms may explain this association. First, myocardial dysfunction is common after cardiac arrest (26, 27). FO may not only exacerbate myocardial dysfunction but also worsen prognosis due to its intolerance. The activation of neurohormones and the renin-angiotensin-aldosterone system (RAAS) leads to systemic and pulmonary artery stiffness, pulmonary hypertension, and right ventricular failure. Positive FB may also directly affect neurological function through various mechanisms, including exacerbating brain edema, disrupting microcirculation, causing endothelial dysfunction, or inducing metabolic imbalances (28). Finally, FO may adversely affect multiple organ systems, including the kidneys and respiratory function, leading to poor outcomes in critically ill patients. FO also affects respiratory function. Patients with acute respiratory distress syndrome (ARDS) in the positive FB group have a significantly higher incidence compared to those in the negative FB group. Studies have shown that FO increases the occurrence of respiratory complications, prolongs mechanical ventilation time, and extends ICU hospitalization time (29, 30).

Fluid management strategies not only emphasize fluid resuscitation but also focus on reducing fluid input after hemodynamic improvement to avoid positive FB (11). Strict fluid management has significant advantages in cardiac arrest treatment. First, after cardiac arrest, the circulatory system undergoes severe damage, and FB is easily disrupted. Strict fluid management can effectively reduce preload, decrease myocardial wall stress, and prevent FO and high hydrostatic pressure. Second, strict fluid management helps maintain colloid osmotic pressure within blood vessels. During the pathophysiological process of cardiac arrest, vascular permeability may increase, causing fluid leakage into the interstitial spaces. By properly controlling the type and amount of fluid, excessive fluid leakage can be prevented, and tissue edema can be reduced. Finally, strict fluid management can modulate the inflammatory response and oxidative stress after cardiac arrest. Excessive fluid input may lead to dilutional coagulopathy and immune suppression, while strict fluid management can avoid these adverse effects. In clinical practice, strict fluid management can be individually tailored based on the patient’s specific condition. Patients with different causes, ages, and underlying diseases have varying fluid requirements after cardiac arrest. Hemodynamic monitoring can help strictly avoid unnecessary fluid input, balancing fluid requirements, fluid responsiveness, and fluid tolerance, thereby reducing the need for additional fluid resuscitation and transfusion (8).

To our knowledge, this is the first retrospective cohort study on the impact of longitudinal FB patterns in CA patients. By applying the GBTM method, we categorized the dynamic changes in FB in CA patients, which may help implement targeted care and early interventions for high-risk groups in clinical practice. Furthermore, the results of multiple subgroup and sensitivity analyses were consistent with the primary analysis, further reinforcing the current study’s conclusions. However, this study has several limitations that should be addressed. First, the MIMIC-IV database is a single-center data source, which may introduce selection bias and lack external validation from other databases. Second, although we adjusted for confounding factors as much as possible, some clinically relevant factors, such as the timing of fluid administration, echocardiography, or specific resuscitation protocols, were not available in the dataset and may still affect the conclusions. Third, to track FB changes in CA patients, we excluded patients admitted to the ICU for less than 3 days, which reduced the sample size and may have underestimated the effect of trajectory indicators on mortality. It is important to note that this exclusion could introduce survivor bias, as patients with shorter ICU stays may have different characteristics or outcomes compared to those with longer stays. Fourth, this observational study can only suggest an association between FB trajectories and CA survival but cannot establish causal relationships between FB and outcomes.

## 5 Conclusion

This study explored the relationship between FB trajectories and 30-day mortality in CA patients. Using the GBTM method, we identified four distinct FB change trajectories, with the rapid shift to negative balance group at medium levels being associated with higher survival rates. Moreover, FO was found to be associated with an increased mortality risk in CA patients. Monitoring FB trajectories may help identify high-risk individuals and regularly assess daily fluid status, along with limiting FO, is crucial for the recovery of ICU CA patients.

## Data Availability

The original contributions presented in the study are included in the article/Supplementary material, further inquiries can be directed to the corresponding author.
