# Supplementary figures and images for: The association between fluid balance trajectories and prognosis in ICU patients with cardiac arrest, a group-based trajectory model analysis
Source: Front Nutr. 2025 Sep 1;12:1664640. doi: 10.3389/fnut.2025.1664640 (PMC12434594; doi:10.3389/fnut.2025.1664640)

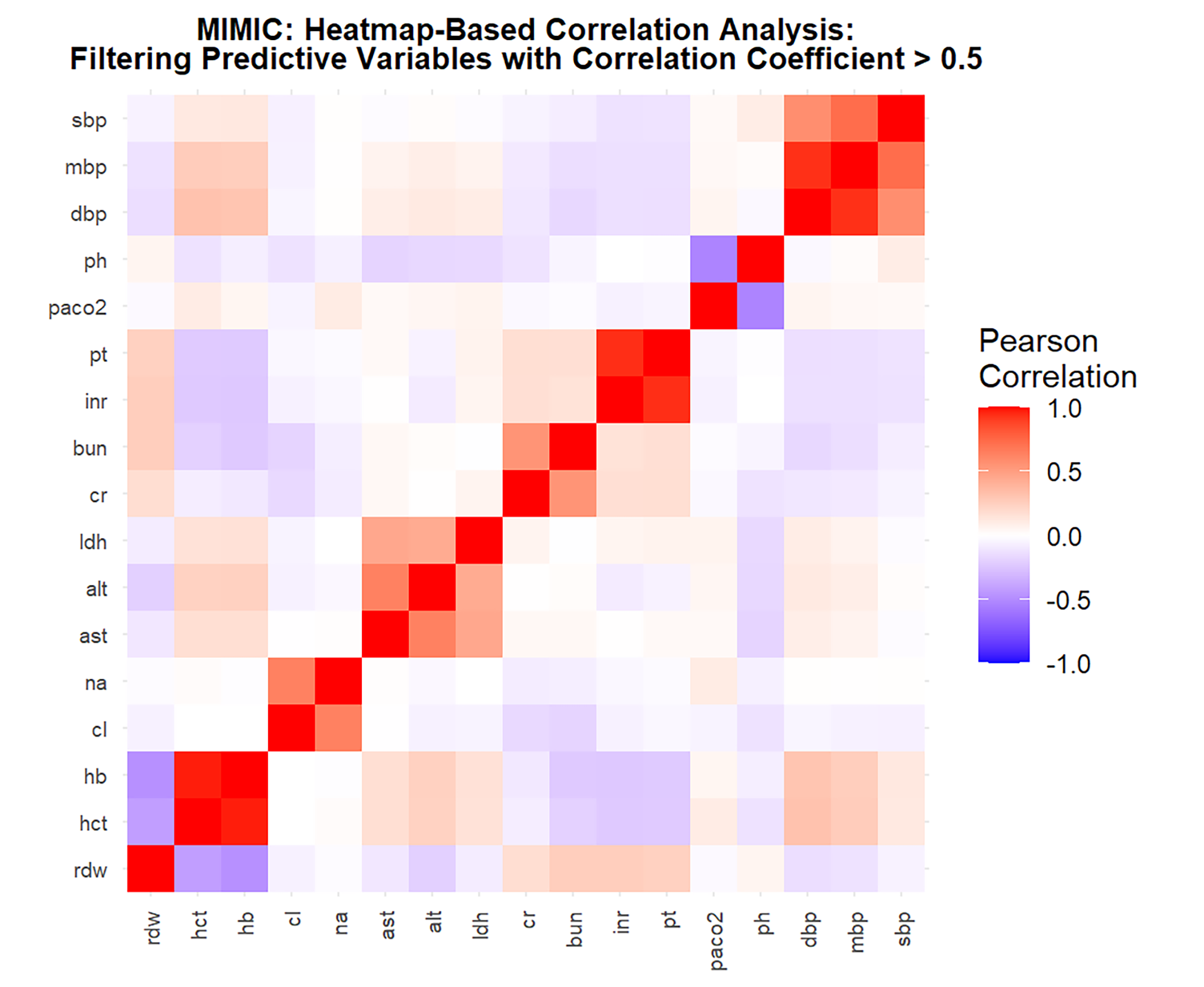

Supplement: Supplementary file 2 [file Image_2.TIFF]
